# Case Report: A Durable Response to Camrelizumab and Apatinib Combination Therapy in a Heavily Treated Small Cell Carcinoma of the Ovary, Hypercalcemic Type

**DOI:** 10.3389/fonc.2022.916790

**Published:** 2022-07-12

**Authors:** Guiling Li, Yao Jiang

**Affiliations:** Cancer Center, Union Hospital, Tongji Medical College, Huazhong University of Science and Technology, Wuhan, China

**Keywords:** small cell carcinoma of the ovary hypercalcemic type, anti-PD-1 antibody, antiangiogenic drug, SMARCA4 mutation, next-generation sequencing

## Abstract

Small cell carcinoma of the ovary, hypercalcemic type (SCCOHT) is a rare and highly aggressive malignancy with a poor prognosis. Most patients experience recurrence even after surgery and chemotherapy, and there are no standard treatment options for recurrent disease. Here, we report the case of a 36-year-old woman with SCCOHT who underwent primary cytoreductive surgery without adjuvant chemotherapy and remained disease-free for 9 months. She then developed retroperitoneal lymph node metastasis and was treated with two cycles of bleomycin/etoposide/cisplatin chemotherapy. However, the disease progressed and the patient received four cycles of liposomal doxorubicin/ifosfamide chemotherapy, followed by local radiation to the enlarged retroperitoneal lymph nodes. She achieved partial remission for 13 months, after which the disease progressed again. Tumor tissues and blood samples were sent for next-generation sequencing. The results indicated a somatic SWI/SNF-related, matrix-associated, actin-dependent regulator of chromatin, subfamily A, member 4 (*SMARCA4*) mutation, microsatellite stability, and a tumor mutation burden of 1.0 muts/Mb without any germline mutations. An anti-PD-1 antibody, camrelizumab, and an antiangiogenic agent, apatinib, were administered, and the patient achieved partial remission for 28 months. Our study provides the first clinical evidence that the combination therapy of camrelizumab and apatinib could be an effective treatment for recurrent SCCOHT.

## Introduction

Small cell carcinoma of the ovary, hypercalcemic type (SCCOHT) is a rare and highly aggressive cancer that accounts for less than 0.01% of all ovarian neoplasms (1). SCCOHT is more prevalent in young women, with a median age at diagnosis of 24 years (2). Clinically, approximately 60% of patients with SCCOHT present with hypercalcemia (3). The initial treatment involves cytoreductive surgery and adjuvant chemotherapy. Several studies have suggested that initial dose-intensive chemotherapy and radiotherapy may extend survival (4–7). However, most patients with SCCOHT experience recurrence, and treatment options are limited for recurrent disease. Therefore, exploration of new treatment strategies is of great importance.

As the development of genomic sequencing, loss-of-function mutations in SWI/SNF-related, matrix-associated, actin-dependent regulator of chromatin, subfamily A, member 4 (*SMARCA4*) have been identified in over 90% of SCCOHT (8–12). This specific genomic change enables new therapeutic opportunities. Several clinical trials have reported modest antitumor efficacy of enhancer of zeste homolog 2 (EZH2) inhibitors in SCCOHT (13, 14). In addition, a series of preclinical studies have demonstrated antitumor activities of histone deacetylase (HDAC), cyclin-dependent kinase 4/6 (CDK4/6), and KDM6 inhibitors in SMARCA4-deficient SCCOHT cells and xenografted models (15–17). Importantly, a positive association between SMARCA4 deficiency and response to immune checkpoint inhibitors (ICIs) has been revealed (18). Herein, we report the case of a patient with refractory SCCOHT relapsed after second-line chemotherapy and radiotherapy, who was successfully treated with the combination of the PD-1 inhibitor, camrelizumab and the antiangiogenic agent, apatinib.

## Case Presentation

**Figure 1:**
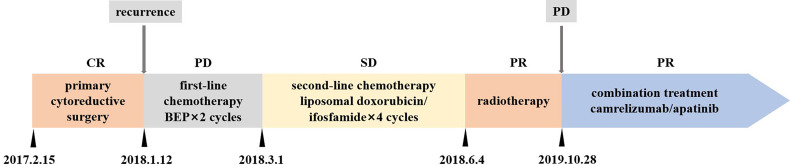
Treatment timeline. CR, complete response; PR, partial response; SD stable disease; PD, progression disease; BEP, bleomycin, etoposide, and cisplatin.

On February 22, 2018, a 36-year-old woman visited the Cancer Center, Union Hospital of Huazhong University of Science and Technology (Wuhan, China) for enlarged retroperitoneal lymph nodes. The treatment timeline is shown in **Figure 1**. The patient had undergone debulking surgery for FIGO stage IA SCCOHT in another hospital on February 15, 2017. She denied any family history of cancer. The serum calcium, cancer antigen 125 (CA125), and neuron-specific enolase (NSE) values were within normal range. Nine months later, a follow-up abdominal computed tomography (CT) scan showed enlarged retroperitoneal lymph nodes. Subsequently, an ultrasonography-guided core needle biopsy followed by pathological examination indicated disease recurrence. The patient was then treated with two cycles of chemotherapy consisting of bleomycin, etoposide, and cisplatin (BEP). However, the retroperitoneal lymph nodes were aggravated. The patient was then admitted to our hospital. She underwent four cycles of liposomal doxorubicin/ifosfamide chemotherapy and maintained stable disease (SD). Subsequently, intensity-modulated radiation therapy to the retroperitoneal lymph nodes was administered; the target volume was administered at 66 Gy in 33 fractions. In addition, 12 Gy in two fractions to the target was delivered using CyberKnife radiosurgery technology and partial response (PR) was achieved.

**Figure 2:**
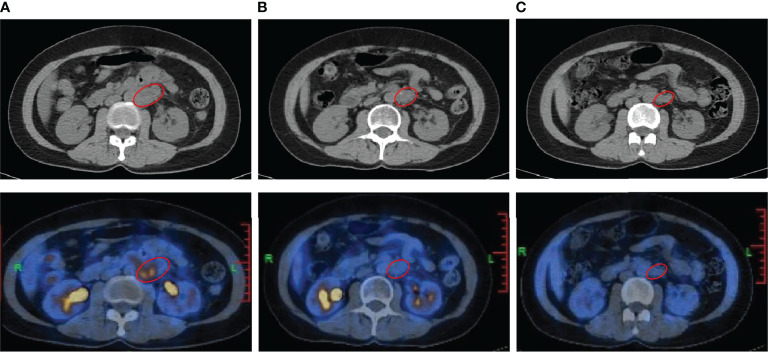
Radiological imaging of the patient before and after the combination treatment of camrelizumab and apatinib. **(A)** ^18^F-FDG positron emission tomography (PET)/CT performed on October 23, 2019, showing recurrence before treatment (baseline). **(B, C)** PET/CT performed on October 9, 2020 **(B)** and November 16, 2021 **(C)** showing the reduced tumor size and SUVmax at 12 months and 25 months after the initiation of treatment, respectively. The red circle indicates the tumor.

Thirteen months later, disease progressed again. A follow-up ^18^F-FDG positron emission tomography (PET)/CT imaging indicated enlarged retroperitoneal lymph nodes measuring 3.9 × 3.7 cm on October 23, 2019 (**Figure 2A**). The maximum standardized uptake value (SUVmax) of the tumor was 8.4. As the patient was resistant to chemotherapy, precision therapy was considered. Biopsied specimens and paired peripheral blood samples were sent for next-generation sequencing using a 520-gene panel (Burning Rock Biotech, Guangzhou, China). The results revealed a somatic *SMARCA4* exon 5 nonsense mutation (c.823C>T, p.Q275*, 95.87%), which was a pathogenic mutation predicted to cause truncation of the SMARCA4 protein. The results also demonstrated microsatellite stability and a tumor mutation burden (TMB) of 1.0 muts/Mb. No germline mutations were detected. In addition, the tumor specimen was tested negative for PD-L1 expression (clone MXR003, MXB biotechnologies, Fuzhou, China).

Recently, studies have suggested a positive association between inactivating mutations in *SMARCA4* and response to ICIs (18, 19). Thus, the anti-PD-1 antibody, camrelizumab was suggested as an effective treatment option. Furthermore, antiangiogenic therapy has been reported to have synergistic antitumor activity with PD-1 inhibitors (20, 21). Therefore, given the negative PD-L1 expression and low TMB level, a combination therapy consisting of camrelizumab and apatinib, an antiangiogenic agent, was administered. The patient commenced treatment with 200 mg intravenous camrelizumab every 3 weeks together with 250 mg oral apatinib once daily from October 28, 2019. After four cycles of treatment, the patient achieved PR, with the largest diameter of the tumor decreasing in size from 3.9 cm to 2.7 cm. Follow-up PET/CT scans at 12 months (**Figure 2B**) and 25 months (**Figure 2C**) after the initiation of treatment demonstrated a sustained disease remission, with the tumor size of 2.7 × 2.4 cm and 2.7 × 1.9 cm, respectively. The SUVmax of the tumor reduced from 8.4 to 1.8 and 1.4, respectively, which indicated dramatic inhibition of tumor activity. The patient discontinued the combination treatment after 2 years. During treatment, the patient developed grade 1 hypothyroidism. Oral levothyroxine at a dose of 100 µg daily was administered, and her thyroid function indices were restored. The patient also experienced grade 1 leukopenia and shingles, which resolved with supportive treatment and did not cause dosage adjustments or interruptions. She did not experience fatigue, hypertension, hemorrhage, diarrhea, proteinuria, or hepatic toxicity, and maintained a good quality of life.

## Discussion

SCCOHT is a rare, aggressive subtype of ovarian cancer that mainly affects young women. Primary debulking surgery with adjuvant chemotherapy is the standard treatment. Studies have suggested that aggressive therapy may improve survival. A GCIG study of 17 patients with SCCOHT reported that most long-term survivors underwent adjuvant radiotherapy and chemotherapy either concurrently or sequentially (7). Another retrospective study of 47 patients with SCCOHT demonstrated that multi-agent chemotherapy and radiotherapy were associated with a better prognosis (4). In a prospective study of 27 patients with SCCOHT treated with first-line intensive chemotherapy, 1-year and 3-year overall survival were 58% and 49%, respectively (5). However, most patients eventually relapse. Currently, there is no consensus on the optimal treatment strategy for recurrent disease. Clinically, chemotherapy and radiation are often administered (7, 22). In this study, the patient underwent BEP chemotherapy after the first relapse. However, the disease progressed. Thus, second-line chemotherapy consisting of liposomal doxorubicin and ifosfamide was administered, followed by local radiation therapy to the enlarged retroperitoneal lymph nodes. The patient achieved PR and remained progression-free for 13 months until she experienced a second recurrence in the same area. As the patient was not sensitive to chemotherapy, precision treatment was considered. The next-generation sequencing results revealed a somatic pathogenic *SMARCA4* mutation.

*SMARCA4*, a core catalytic component of the SWI/SNF complex, has been identified as a tumor suppressor (23). Inactivating *SMARCA4* mutations have been found in the majority of patients with SCCOHT (10). A growing body of evidence suggests that inactivation of *SMARCA4* promotes the oncogenic activities of EZH2 through transcriptional repression caused by aberrant H3K27me3 (24, 25). Therefore, EZH2 inhibitors have been tested in SMARCA4-deficient tumors. In a phase I study, the selective EZH2 inhibitor tazemetostat demonstrated a clinical benefit in SCCOHT (one PR and one SD) (13). In another phase II study, tazemetostat induced one PR among ten patients with SCCOHT (14). Taken together, EZH2 inhibitors demonstrate only modest efficacy in SCCOHT. In addition, HDAC inhibitors display synergistic antitumor activity with EZH2 inhibitors in SCCOHT cells and xenografted tumors (15). In a recent study, the KDM6 inhibitor exhibits significant antitumor efficacy in SCCOHT mice model (16). Additionally, several studies have conducted siRNA screens to identify the kinase dependencies of SMARCA4-deficient SCCOHT cells, and demonstrated that bromodomain and extra-terminal motif containing proteins (BETs), and CDK4/6 inhibitors, as well as ponatinib were effective antitumor agents for SCCOHT both *in vitro* and *in vivo* (17, 26–28). However, these are mainly preclinical studies, and further research is needed.

Recently, studies have suggested a positive association between inactivating mutations in *SMARCA4* and response to ICIs. A clinical study showed that patients with SMARCA4- deficient non-small cell lung cancer were more sensitive to ICIs compared with *SMARCA4*-wild type (24). Jelinic et al. reported that eight out of eleven SCCOHT cases displayed high PD-L1 expression and strong T-cell infiltration, despite having a low mutation burden. Furthermore, a case series of four patients with recurrent SCCOHT responded to PD-1 inhibitors (18). Interestingly, three of them were treated with local radiation followed by PD-1 inhibitors. Radiation therapy has been shown to induce tumor antigen release, promote the priming and effector phases of the antitumor T-cell response, and thereby enhance antitumor immune response (29). Clinical studies demonstrated that patients who received radiotherapy prior to ICIs exhibited superior therapeutic response than those who received ICIs only (30, 31). Therefore, it can be hypothesized that the response of these patients may be partially due to the synergetic antitumor activity of radiation therapy. In a recent study, a patient with refractory SCCOHT achieved PR after four cycles of combination treatment of nivolumab with ipilimumab, and subsequently recurred two months after single nivolumab maintenance (32). Thus, combination immunotherapy was considered for this patient.

Camrelizumab is a humanized, monoclonal antibody that blocks PD-1. It has been approved for the treatment of a range of malignancies in China (33). Antiangiogenic drugs have been reported to increase the infiltration of immune cells, improve the immune-suppressed microenvironment, and thereby enhance the efficacy of ICIs (34). Several clinical trials have demonstrated synergistic antitumor efficacy between ICIs and antiangiogenic agents in various malignancies (35). In the KEYNOTE-426 trial, the combination therapy of pembrolizumab plus axitinib showed improved survival outcomes compared with sunitinib monotherapy in patients with previouly untreated advanced renal cell carcinoma (RCC), regardless of PD-L1 expression (36). In the phase III JAVELIN Renal 101 study, the combination of avelumab and axitinib demonstrated longer progression-free survival (PFS) and higher objective response rate (ORR) than sunitinib in advanced RCC (37). Further biomarker analysis revealed that neither PD-L1 expression nor TMB level differentiated PFS in either study arm (38). In our study, the tumor specimen was tested negative for PD-L1 with low TMB level. Therefore, combination therapy of ICIs and antiangiogenic agents was considered. Apatinib is a small-molecule tyrosine kinase inhibitor that selectively inhibits vascular endothelial growth factor receptor-2, and also mildly suppress the activities of Ret, c-kit, and c-src (39). This agent has demonstrated antitumor activity in a series of solid tumors (40). Furthermore, several studies have demonstrated that apatinib improves antitumor efficacy of camrelizumab (41, 42). In a small cohort of 11 patients with heavily treated non-small cell lung cancer, co-administration of apatinib and camrelizumab achieved an ORR of 55.6% (41). Thus, combination therapy of camrelizumab and apatinib was administered, and the patient achieved PR after four cycles of treatment. She remained on camrelizumab and apatinib for a total of 2 years and continued to have a sustained partial response. Currently, a clinical trial of the PD-1 inhibitor pembrolizumab with chemotherapy for the treatment of SCCOHT is ongoing (NCT04602377, recruiting). Our case indicates that the combination therapy of camrelizumab and apatinib could be effective for patients with recurrent SCCOHT.

## Conclusion

In conclusion, our study provides the first clinical evidence that the combination of camrelizumab and apatinib could be an effective treatment for patients with recurrent SCCOHT. Additional studies are needed to further investigate this treatment strategy in patients with SCCOHT.

## Data Availability Statement

The original contributions presented in the study are included in the article/supplementary material. Further inquiries can be directed to the corresponding author.

## Ethics Statement

The studies involving human participants were reviewed and approved by the Ethics Committee of Union Hospital of Huazhong University of Science and Technology (20220023). The patients/participants provided their written informed consent to participate in this study. Written informed consent was obtained from the individual(s) for the publication of any potentially identifiable images or data included in this article.

## Author Contributions

YJ: conceptualization; investigation; writing – review and editing. GL: collection of data; writing – original draft. All authors contributed to the article and approved the submitted version.

## Funding

This work was supported by the National Natural Science Foundation of China (grant number 81902854). The sponsors had no role in the design and conduct of the study; the preparation, review, or approval of the manuscript; or the decision to submit the manuscript for publication.

## Author Disclaimer

The content is solely the responsibility of the authors and does not necessarily represent the official views of the sponsors.

## Conflict of Interest

The authors declare that the research was conducted in the absence of any commercial or financial relationships that could be construed as a potential conflict of interest.

## Publisher’s Note

All claims expressed in this article are solely those of the authors and do not necessarily represent those of their affiliated organizations, or those of the publisher, the editors and the reviewers. Any product that may be evaluated in this article, or claim that may be made by its manufacturer, is not guaranteed or endorsed by the publisher.

## Abbreviations

BEP, bleomycin, etoposide, and cisplatin; BETs, bromodomain and extra-terminal motif containing proteins; CA125, cancer antigen 125; CDK4/6, cyclin-dependent kinase 4/6; CT, computed tomography; EZH2, enhancer of zeste homolog 2; HDAC, histone deacetylase; ICIs, immune checkpoint inhibitors; NSE, neuron-specific enolase; ORR, objective response rate; PET, positron emission tomography; PFS, progression-free survival; PR, partial response; RCC, renal cell carcinoma; SCCOHT, small cell carcinoma of the ovary, hypercalcemic type; SD, stable disease; SMARCA4, SWI/SNF-related, matrix-associated, actin-dependent regulator of chromatin, subfamily A, member 4; SUVmax, maximum standardized uptake value; TMB, tumor mutation burden.
